# Mitochondrial Respiratory Complexes as Targets of Drugs: The PPAR Agonist Example

**DOI:** 10.3390/cells11071169

**Published:** 2022-03-30

**Authors:** Patrizia Bottoni, Alessandro Pontoglio, Salvatore Scarà, Luisa Pieroni, Andrea Urbani, Roberto Scatena

**Affiliations:** 1 Dipartimento di Scienze Biotecnologiche di Base, Cliniche Intensivologiche e Perioperatorie, Università Cattolica del Sacro Cuore, Largo Francesco Vito 1, 00168 Rome, Italy; espeedy@libero.it (A.P.); scarsalvatore8@gmail.com (S.S.); andrea.urbani@unicatt.it (A.U.); roberto.scatena@figliesancamillo.it (R.S.); 2 IRCCS-Santa Lucia Foundation, 00179 Rome, Italy; l.pieroni@hsantalucia.it; 3 Dipartimento di Medicina di Laboratorio, Fondazione Policlinico Gemelli, Largo Agostino Gemelli 8, 00168 Rome, Italy; 4 Dipartimento di Medicina di Laboratorio, Madre Giuseppina Vannini Hospital, Via di Acqua Bullicante 4, 00177 Rome, Italy

**Keywords:** mitochondria, complex I (NADH: ubiquinone oxidoreductase), reactive oxygen species (ROS), drug toxicity, therapeutic drug monitoring, cancer

## Abstract

Mitochondrial bioenergetics are progressively acquiring significant pathophysiological roles. Specifically, mitochondria in general and Electron Respiratory Chain in particular are gaining importance as unintentional targets of different drugs. The so-called PPAR ligands are a class of drugs which not only link and activate Peroxisome Proliferator-Activated Receptors but also show a myriad of extrareceptorial activities as well. In particular, they were shown to inhibit NADH coenzyme Q reductase. However, the molecular picture of this intriguing bioenergetic derangement has not yet been well defined. Using high resolution respirometry, both in permeabilized and intact HepG2 cells, and a proteomic approach, the mitochondrial bioenergetic damage induced by various PPAR ligands was evaluated. Results show a derangement of mitochondrial oxidative metabolism more complex than one related to a simple perturbation of complex I. In fact, a partial inhibition of mitochondrial NADH oxidation seems to be associated not only with hampered ATP synthesis but also with a significant reduction in respiratory control ratio, spare respiratory capacity, coupling efficiency and, last but not least, serious oxidative stress and structural damage to mitochondria.

## 1. Introduction

Warburg’s brilliant observation, at the beginning of the twentieth century, that cancer cells—contrary to normal cells—use glycolysis to generate energy even when oxygen is available had a strong impact on research and application in the oncology field, so much so that important clinical applications, as the visualization of tumors by positron emission tomography (PET) imaging technique and therapeutic strategies preferentially targeting cancer cells have been developed on the basis of these acquisitions and are still currently utilized in clinical practice. Specifically, Warburg asserted that this particular metabolic shift characterized by high glucose uptake and elevated lactate production was due to a mitochondrial impairment and was the origin of malignant transformation. It followed that the role of mitochondria in cancer has been neglected for a long time. 

However, in recent years there has been a renewed interest in the study of mitochondria and an extraordinary progress in mitochondrial science, in large part ascribable to the recognition that genetic and/or biochemical alterations in this organelle can contribute to a variety of human diseases, including cancer [1,2]. In light of these findings and other experimental evidence, there has been a significant re-evaluation of the role of the oxidative mitochondrial metabolism in cancer cell growth and progression [3,4,5,6,7,8].

Another interesting aspect to evaluate is the interaction of pharmacological agents with mitochondria, which are becoming drug targets with increasing frequency. Many exhaustive reviews suggest that mitochondria are potential targets of drugs (nucleoside reverse transcriptase inhibitors, anthracyclines, biguanides, fibrates, tetracyclines, some NSAIDs and so on), all pointing out a series of relevant pharmacological and toxicological implications. However, the paucity of experimental data to illustrate this interaction has until now limited their true translational potential [2,9,10,11,12,13]. In fact, the molecular analysis of the interplay between pharmacological agents and mitochondria is an aspect of biochemistry that is too often disregarded, and this inadequate consideration has already caused serious and negligent clinical outcomes [11,14,15,16,17,18]. Importantly, a careful biochemical and pharmacological approach may shed new light on some aspects of the physiology and pathophysiology of mitochondria, not only in terms of pharmacotoxicology but also in terms of aging and age-associated disorders (cancer, neurodegeneration and so on), as examples. 

Interestingly, considering both the important clinical use of well-known class of chemical derivatives of fibric acid, the peroxisome proliferator activated receptors (PPAR) ligands, and the growing role of the new class of their derivatives, the thiazolidinediones (TZDs), in the pathophysiology of various diseases (diabetes, obesity, hepatic dysfunctions, cognitive disorders and, most importantly, cancer) together with the intriguing pharmacotoxicological profiles of their putative ligands, we propose to better delineate the interrelationships existing between mitochondria and PPAR ligands. This is also in light of some dramatic toxic effects that affected organs rich in mitochondria (the muscle, heart, liver, and kidney) following the administration of these drugs [19,20,21,22,23]. This effort will enable not only development and definition of a true therapeutic index of some currently used and future PPAR ligands, but also better understanding of the pharmacotoxicology of mitochondria in general.

Our previous studies showed a dose-dependent derangement of the mitochondrial respiratory chain, specifically at the level of CI, induced by different PPAR ligands in various human cell lines derived from all three germ layers. Importantly, this functional derangement was associated with serious structural damage to these organelles [24,25,26]. In our opinion, drug-induced mitochondrial dysfunction influences the therapeutic index of this class of drugs, both in terms of pharmacological activities and toxicological properties [26,27].

In this study, we use a more accurate and widespread oxygraphic, metabolic and proteomic approach to better define PPAR ligand-induced mitochondrial derangement in HepG2 cells, a human hepatocarcinoma cell line. This well-differentiated hepatocellular carcinoma cell line is a well-established, suitable model to study liver metabolism and environmental, dietary and drug toxicity [28]. The data we derived from using these cells confirm the pathogenic role of this PPAR ligand-induced CI derangement, stressing its influences on some complicated extra-peroxisomal biological activities of these drugs. These activities appear to induce a complex network of damage and compensatory mechanisms that extend their effects to other complexes of the ERC, potentially influencing all mitochondrial activities (oxidative metabolism, ROS production, intracellular signaling, and so on). Moreover, the data highlight some structural characteristics for potential mitotoxicants and, ultimately, point to peculiar compensatory mechanisms related to the derangement in mitochondrial oxygen utilization.

## 2. Materials and Methods

### 2.1. Chemicals and Reagents

All chemicals were from Sigma Chemical Co. (Milan, Italy) unless otherwise indicated. Cell culture media and supplements were obtained from Lonza Group Ltd., Basel, Switzerland.

### 2.2. Cell Culture

The HepG2 human hepatocellular carcinoma cell line was obtained from the Interlab Cell Line Collection (ICLC, National Institute for Cancer Research, Genoa, Italy). The cells were grown at 37 °C under a humidified atmosphere of 5% CO_2_ in Dulbecco’s modified Eagle’s medium (DMEM) with 4.5 g/L glucose, supplemented with 10% (*v*/*v*) heat-inactivated fetal calf serum, 2 mM glutamine, 100 U/mL penicillin, and 100 μg/mL streptomycin (Lonza Group Ltd., Basel, Switzerland). Exponentially growing cells were trypsinized with 0.05% trypsin-0.02% EDTA (Lonza Group Ltd., Switzerland) seeded at 2 × 10^4^ cells/cm^2^ and incubated in media containing various concentrations of drugs.

### 2.3. Drug Treatment

Stock solutions were prepared immediately before use. Bezafibrate, gemfibrozil, clofibric acid and ciglitizone were pre-dissolved in dimethyl sulfoxide (DMSO). The final concentrations of the drugs were bezafibrate, 1 mM; gemfibrozil, 0.23 mM; clofibric acid, 0.7 mM; and ciglitizone at 1, 10, 30 and 50 μM. The final concentration of DMSO, used as the vehicle, was the same in all samples during the experiments (0.1% *v*/*v*).

### 2.4. High-Resolution Respirometry

Respiration in intact and permeabilized HepG2 cells was monitored with high-resolution respirometry (OROBOROS Oxygraph-2k, Innsbruck, Austria) operating at 37 °C with a 2 mL chamber volume [23]. Cellular respiration experiments were carried out in two O2k chambers operated in parallel after calibration of the oxygen sensors at air saturation and an instrumental background correction. Calibration with air-saturated medium was performed immediately before the oxygen flux measurement was taken. The data acquisition and analysis were carried out using DatLab software (OROBOROS Instruments).

#### 2.4.1. Permeabilized Cells

Exponentially growing HepG2 cells were detached, seeded at 2 × 10^4^ cells/cm^2^ and incubated with various concentrations of drugs for 30 min at 37 °C. After the incubation period, the medium was removed, and the cells were suspended at a density of 0.5 × 10^6^ cells/mL in MiR05 (110 mM sucrose; 60 mM K-lactobionate; 0.5 mM EGTA; 3 mM MgCl_2_; 20 mM taurine; 10 mM KH_2_PO_4_; 20 mM HEPES, pH 7.1 at 30 °C; and 0.1% BSA that was essentially fatty acid free) to evaluate the activities of the respiratory complexes. The respiration rates of permeabilized cells were determined using substrate-uncoupler inhibitor titration (SUIT) protocols [29,30,31]. After the measurement of routine endogenous respiration for 15 min was taken, the CI resting state was assessed by the addition of malate (5 mM) and glutamate (5 mM) as the CI substrate supply, followed by cell permeabilization with digitonin (10 μg∙10^−6^ cells Dig), and then, CI active (state 3) was assessed by the addition of ADP (4.8 mmol/L). The addition of succinate (9.5 mmol/L) provided state 3 respiration data with information on parallel electron input to complexes I and II (the CI-II active states). The integrity of the outer mitochondrial membrane was established by the addition of cytochrome c (19 μmol/L). After inhibition by oligomycin (2 μg/mL, the LEAK state), a protonophore, carbonyl cyanide p-trifluoro-methoxyphenyl hydrazone (FCCP, 1.2 µM), was added to study the ETS capacity (the ETS state). The addition of rotenone (0.5 μM) resulted in inhibition of CI for the examination of O_2_ flux with the CII substrate alone, while antimycin A (2.5 μM) was added to inhibit CIII and to observe non-mitochondrial respiration with small contributions from electron leakage in the uncoupled state.

#### 2.4.2. Intact Cells

HepG2 cells were incubated at a density of 0.5 × 10^6^ cells/mL in a 2 mL DMEM glass chamber at 37 °C and thereafter investigated using a phosphorylation control protocol [29]. Cellular respiration was first measured in DMEM until it reached a steady state, at which point the respiratory flux is constant, defined as BASAL. After observing the basal flux for 10 min, different drugs or the vehicle were added to the cellular suspension in the chamber. The resulting respiratory flux, followed for 30 min, is termed the ROUTINE respiration state (R state) and corresponds to the basal respiration in the presence of DMSO or drugs dissolved in DMSO (in this case, ciglitizone, bezafibrate, gemfibrozil and clofibric acid). After incubation with the drug, ATP synthase was inhibited by the addition of oligomycin (2 μg/mL) added to each chamber to detect the oligomycin-inhibited leak rate of respiration (L state). This L state is caused mainly by the compensation for proton leakage from the mitochondrial intermembrane space after the inhibition of ATP synthase [29,30]. The maximal capacity of the ETS was obtained by the addition of small volumes of FCCP and instantaneous observation of its effect on cellular respiration to achieve maximum mitochondrial respiration in the uncoupled state. Subsequently, cellular respiration was measured in the presence of rotenone (0.5 μM), which selectively inhibits CI, and then in the presence of antimycin A (2.5 μM), which inhibits CIII, to estimate residual oxygen consumption (ROX). In addition to instrumental background, the mitochondrial respiration was corrected for the oxygen flux due to ROX [28,29]. The rate of mitochondrial ATP synthesis (oligomycin-sensitive respiration) was calculated as the difference between ROUTINE respiration and LEAK respiration according to Gnaiger [30].

#### 2.4.3. Respiratory Control Ratios

In addition to information on absolute respiratory fluxes, the respiratory acceptor control ratio (RCR) and the spare respiratory capacity (SRC) were derived from the experimental protocols used (https://wiki.oroboros.at/index.php/MitoPedia:_Respiratory_control_ratios (accessed on 28 March 2021) [31]). In intact cells, the L/E and R/E ratios were also calculated: the L/E coupling control ratio combines the effects of coupling and ETS capacity, and the R/E coupling control ratio reflects the combined effects of cell physiological state, coupling, OXPHOS capacity and excess capacity (https://wiki.oroboros.at/index.php/MitoPedia:_Respiratory_states (accessed on 28 March 2021) [24]). SRC was measured by the difference between ETS respiration and routine respiration. The coupling efficiency was determined from the change in basal respiration rate upon addition of oligomycin [31].

### 2.5. Assay for ROS

Intracellular ROS were analyzed using the fluorescent probe carboxy-2′-7′-dichlorofluorescein-diacetate (DCF-DA) as previously described [32]. Treated and untreated cells were washed twice and loaded with 5 μM DCF-DA (dissolved in DMSO) for 30 min at 37 °C in the dark. Fluorescent units were measured using a CytoFluor 2300/2350 fluorescence measurement system (Millipore Corp., Bedford, MA, USA) at 504 nm excitation and 529 nm emission. Background fluorescence was subtracted from the measured values.

### 2.6. Proteomic Analysis

#### 2.6.1. Protein Purification and Label-Free Differential Proteomic Shotgun Analysis

The analysis was performed on total proteins extracted from HepG2 cells treated or not treated with 50 µM CGZ for 96 h. Cell pellets were suspended in denaturing buffer (6 M urea in 100 mM Tris-Cl, pH 7.8) and manually fractionated with a mini potter. The samples were then sonicated in a water bath (Ultrasonic Cleaner, CP104, EIA) at 95% US power at room temperature for 15 min and centrifuged at 13,000 rpm at 4 °C for 30 min. Protein concentrations were determined for each supernatant. Twenty-five micrograms of total protein solution were denatured with 10 mM DTT and alkylated with 20 mM IAA prior to trypsin digestion (the enzyme:proteins ratio was 1:50) for 16 h at 37 °C. Digestion was interrupted by acidification with 0.1% FA. Upon digestion, 0.125 μg/μL of tryptic peptide solution spiked with 200 fmol/μL yeast enolase digestion standard (SwissProt P00924) (Waters, Milford, MA, USA) was used for label-free differential proteomic shotgun analysis by means of an ultra-performance liquid chromatography system coupled to a hybrid quadruple orthogonal acceleration time-of-flight mass spectrometer (nUPLC-Q-IMS-TOF–CID/ETD, Synapt G2-si (Waters)). In particular, instrumental triplicates were acquired for each sample on a MClass nUPLC™ (Waters Corp.) chromatographic system, with a 15 cm C18 capillary column with a particle size of 1.7 µm. Mass spectrometry data were acquired in MS^E^ (expression mode: data-independent parallel parent and fragment ion analysis). The acquired LC-MS/MS data were then processed by ProteinLynx Global SERVER v3.0.2 (PLGS) (Waters Corp.), software that enables the simultaneous identification and relative quantification of protein expression under different conditions. The proteins were qualitatively identified through searching a human database (UniProt KB/Swiss-Prot Protein Knowledgebase release 2016_02 of 17-Feb-16, containing 5,550,552 sequence entries, taxonomical restrictions: Homo sapiens, 20,198 sequence entries) to which data from *Saccharomyces cerevisiae* enolase (accession number: P00924) were appended. The identified proteins were normalized against the P00924 data in the differential quantitative analysis (expression analysis). Our data were qualified to detect a difference in relative ratios larger than 20%; however, to keep a conservative approach, we filtered the protein hits to include only those with a fold difference larger than 30% (corresponding to a ratio of + or −1.3). These performance criteria are in line with those reported in previous papers from our group and others [33].

#### 2.6.2. Bioinformatics Analysis

Gene Ontology annotations were obtained using the PANTHER online tool (version 11.1, http://www.pantherdb.org/ (accessed on 5 February 2020)) with the entire *Homo sapiens* genome serving as the reference set, and the functional classifications were obtained as a gene list that was analyzed to obtain the classification of differentially expressed proteins according the following categories: family and subfamily, molecular function, biological process, and pathway.

For the protein network analysis, the data sets obtained from shotgun proteomics, including the gene identifier and relative expression values (fold-change treated/not treated) of each protein, were uploaded to QIAGEN Ingenuity Pathway Analysis software (IPA, QIAGEN Redwood City, www.qiagen.com/ingenuity, build version: 389077M; content version: 27216297; Release Date: 16 March 2016; www.ingenuity.com) for core analysis applications [34].

### 2.7. Statistical Analysis

All values are expressed as the means ± SEM of three experiments, completed in duplicate. The group means were compared by analysis of variance (ANOVA) followed by a multiple comparison of the means by a Dunnett test. For all statistical evaluations, a *p* value of less than 0.05 was considered significant.

## 3. Results

### 3.1. High-Resolution Respirometry (HRR) of Permeabilized Cells

To assess the effect of different drugs on mitochondrial respiration, HepG2 cells were incubated in vitro with some so-called PPAR-alpha ligands and increasing concentrations of ciglitizone, a PPAR-gamma ligand, according to Gnaiger’s method for permeabilized cells [29].

**Figure 1:**
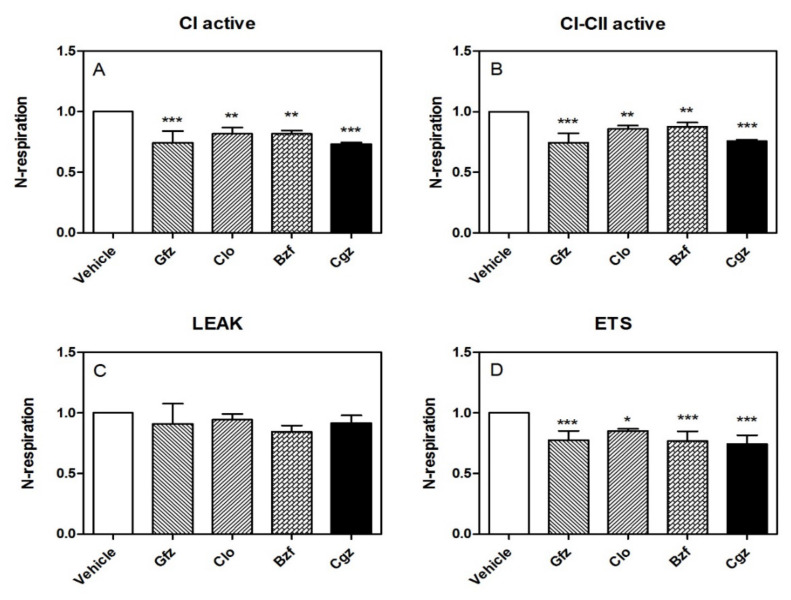
The effect of different PPAR ligands on CI and the respiratory states in permeabilized HepG2 cells. Respiration (indicated as the rate of oxygen flux normalized to the respective control) was evaluated at different stages. Panel (**A**): CI-active, CI respiration activated by excess ADP; Panel (**B**): CI/II-active, combined CI and CII respiration; Panel (**C**): Leak respiration, induced by the inhibition of ATP synthase by oligomycin; and Panel (**D**): ETS respiration, recorded in presence of the optimal FCCP concentration. Statistical analyses were performed with one-way ANOVA, followed by a multiple comparison of the means by a Dunnett test to calculate significance: * *p* < 0.05, ** *p* < 0.01, and *** *p* < 0.001.

Importantly, the HRR data confirmed the iatrogenic CI derangement in its active state (obtained by adding glutamate/malate and ADP). Specifically, the reduction in O_2_ consumption was approximately −27% with respect to the control, *p* < 0.001, for ciglitizone; for gemfibrozil it was −26%, *p* < 0.001; for clofibric acid it was −15%, *p* < 0.01; and for bezafibrate it was −16%, *p* < 0.05 (Figure 1A). Adding succinate, a substrate for CII, the inhibition of cellular respiration did not show significant modifications compared with the inhibition reported for CI: for ciglitizone it was approximately −24%, *p* < 0.001; for gemfibrozil it was −26%, *p* < 0.001; for clofibric acid it was −14%, *p* < 0.01; and for bezafibrate it was −12%, *p* < 0.05) (Figure 1B). Interestingly, the inhibition of ATP synthase by oligomycin, which significantly reduces electron flow through the electron transport chain, did not indicate significant modifications to leak respiration in terms of O_2_ consumption in the permeabilized cells treated with different drugs compared to that in the controls (Figure 1C).

Notably, the level of maximal uncoupled respiratory activity, a measure of the respiratory ETC recorded in the presence of optimal uncoupler (FCCP) concentrations, was negatively influenced by these drugs with respect to the controls. Specifically, we measured the following effects at the specified drug concentrations: ciglitizone −18% (*p* < 0.001), gemfibrozil −23% (*p* < 0.001), clofibric acid −15% (*p* < 0.01), and, finally, bezafibrate −16% (*p* < 0.05). This trend showed a strict relation to CI drug-induced dysfunction (Figure 1D).

**Figure 2:**
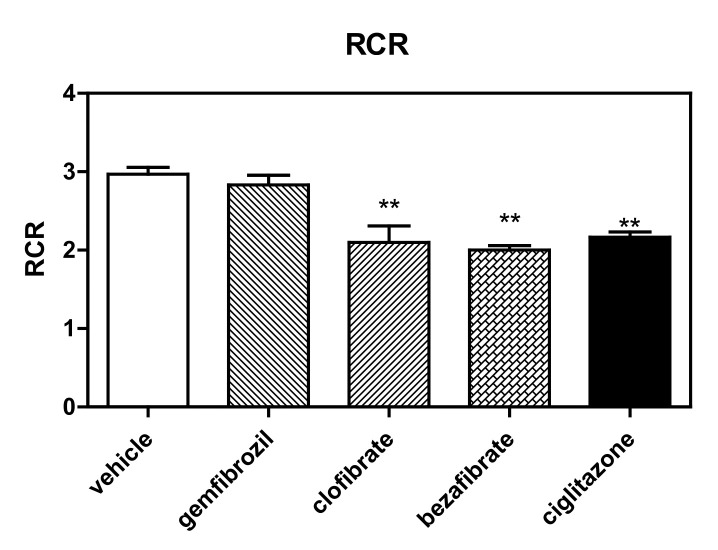
RCR of permeabilized HepG2 cells treated with different PPAR ligands. ** *p* < 0.01.

Finally, the best evidence of ERC derangement is the modification of RCR (ADP/no ADP) induced by these molecules (Figure 2), evaluated according the methods of both Gnaiger [29,30] and Brand and Nicholls [31] for permeabilized cells. Interestingly, all drugs were demonstrated to modify RCR (mainly due to derangement in state 3). Specifically, the measured RCR reduction levels were as follows with respect to the controls: ciglitizone −27% (*p* < 0.01), gemfibrozil −7% (*p*: NS), clofibric acid −29% (*p* < 0.01), and bezafibrate −33% (*p* < 0.01).

### 3.2. HRR of Intact Cells

**Figure 3:**
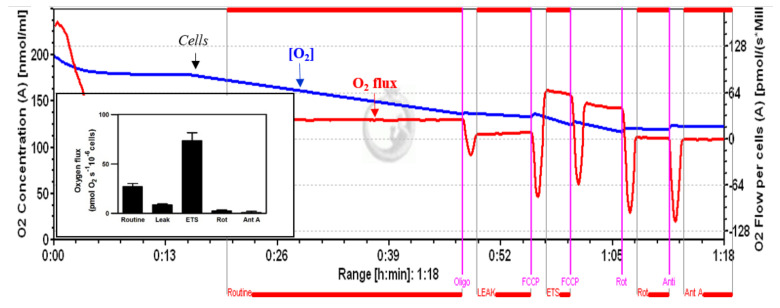
A typical profile of cellular respiration of untreated HepG2 cells, recorded by high-resolution respirometry, with traces of oxygen concentration (blue line) and oxygen flux (red line), corrected for instrumental background. Vertical lines represent responses to additions of oligomycin (Oligo), which reduced respiration to the LEAK state (inhibition of ATP synthase); FCCP, which stimulated respiration in the non-coupled state of the ETS; a second FCCP titration illustrates the inhibition by excess uncoupler concentration; and inhibition by rotenone (Rot) and antimycin A (Ant A).

The respirometry measurements in intact cells seem to have greater physiological relevance. In fact, in this experimental protocol, the interactions/influences of mitochondrial perturbation on the cell are preserved [31,35,36]. Figure 3 shows a typical profile of the cellular respiration in untreated HepG2 cells, recorded by HRR. Under routine conditions, the HepG2 cells showed an oxygen consumption of 26.9 ± 3.2 pmol∙s^−1^/10^−6^ cells. Inhibition by oligomycin A and rotenone reduced this respiration rate to 8.5 ± 1.09 and 2.39 ± 1.03 O_2_ pmol∙s^−1^∙10^−6^ cells, while uncoupling by FCCP increased the respiratory rate to 73.36 ± 8.1 pmol∙s^−1^/10^−6^ cells, thus indicating an increase of approximately 170%, with respect to routine respiration.

The inset shows a bar graph of the different phases of oxygen consumption.

PPAR ligands modified the oxygen consumption profile of these cells. Importantly, this reduced oxygen flux showed a trend partially different from that observed both with spectrophotometric approach [25,26,27,37] and permeabilized cells, above all in terms of routine respiration.

**Figure 4:**
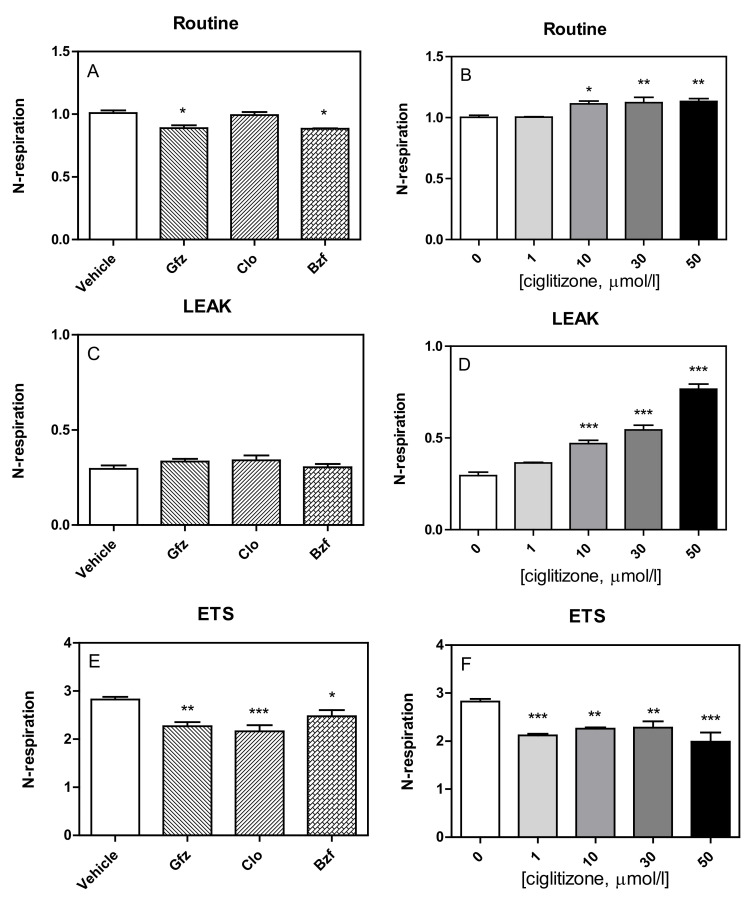
The effect of PPAR ligands (on the left) and increasing concentrations of ciglitizone (on the right) on the respiratory states in HepG2 cells. Cellular respiration, calculated as the oxygen flux, ROX-corrected, normalized to the respective basal rate) was evaluated at different stages: Routine (Panel **A**,**B**), Leak (Panel **C**,**D**), ETS (Panel **E**,**F**). Data show the mean ± SEM of three experiments in duplicate, analyzed by ANOVA, using Dunnett multiple comparison test to calculate significance (* *p* < 0.05, ** *p* < 0.01, and *** *p* < 0.001).

In fact, the respiratory fluxes in HepG2 cells, after correction for instrumental background, showed that the PPAR ligands had slightly modified routine respiration, as well as alterations in the other respiratory states (Leak, ETS) (Figure 4).

Specifically, the tested drugs had a different influence on the routine respiration of HepG2-intact cells (Figure 4A,B). All PPAR ligands seemed to increase oxygen consumption during the leak state (gemfibrozil: +14%, *p* = NS; clofibric acid: +17%, *p* = NS; and bezafibrate: +4%, *p* = NS) with respect to the controls, but only with ciglitizone was this increase significant (1 µmol: +24%, *p* = NS; 10 µmol: +62%, *p* < 0.001; 30 µmol/L: + 86%, *p* < 0.001; 50 µmol/L: +165%, *p* < 0.001) (Figure 4C,D).

Similar to those of permeabilized cells, the maximal oxygen consumption rates of intact cells, corrected for ROX, after FCCP addition, which reflects ETS capacity, were negatively influenced by these drugs (GFZ, CLO, and BZF: −19%, *p* < 0.01; −23%, *p* < 0.01; and −12%, *p* < 0.05; respectively). Similarly, ciglitizone was also shown to inhibit ETS capacity of HepG2 cells, reaching 29% with 50 µmol/L (Figure 4E,F).

**Figure 5:**
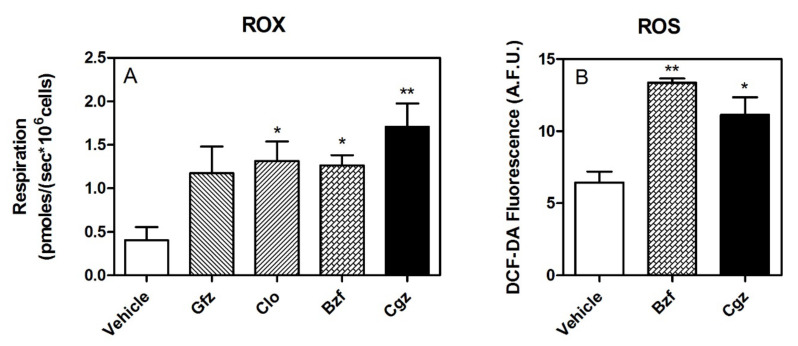
ROX and ROS. Panel (**A**): Residual oxygen consumption (ROX), measured after inhibition of the ERC in treated and untreated HepG2 cells. Panel (**B**): Intracellular ROS levels in the HepG2 cells upon treatment with 50 μM CGZ and 1 mM BZF. ROS data, expressed as arbitrary fluorescence units (A.F.U.), are the means ± SEM of four replicates from each condition (* *p* < 0.05 and ** *p* < 0.01).

Importantly, ROX was significantly increased by drug treatment, even a few picomoles (Figure 5A). Moreover, pharmacological treatment seems to also be associated with a release of higher level of ROS, as shown by the measurement of DCF-DA fluorescence (Figure 5B).

**Figure 6:**
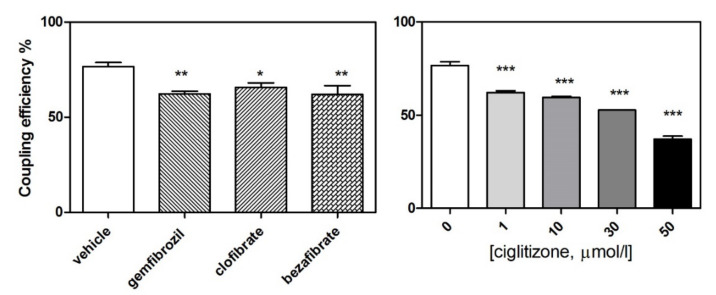
Coupled respiration (expressed as a percentage) in the HepG2 cell cultures treated with different PPAR ligands (**on the left**) and with increasing concentrations of ciglitizone (**on the right**) (* *p* < 0.05, ** *p* < 0.01, and *** *p* < 0.001).

Notably, drug treatment induced a significant reduction in coupled respiration (oligomycin-sensitive respiration/basal respiration), corrected for ROX, in the HepG2 cells: gemfibrozil: −23%, *p* < 0.01; clofibric acid: −11%, *p* = NS; and bezafibrate: −16% *p* = NS. Similarly, ciglitizone deranged coupled respiration in a dose-dependent manner: 1 µmol: −19%, *p* < 0.001; 10 µmol: −23%, *p* < 0.001; 30 µmol/L: −31%, *p* < 0.001; and 50 µmol/L: −52%, *p* < 0.001) (Figure 6).

**Figure 7:**
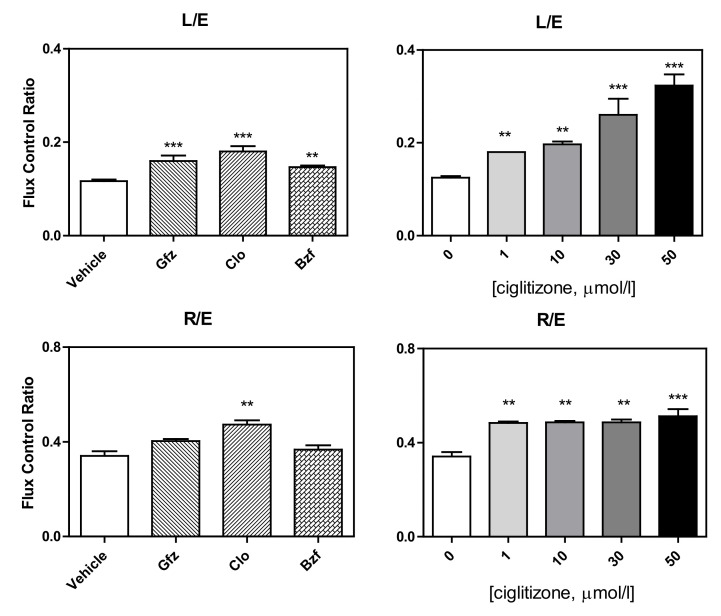
Respiratory control ratios. LEAK flux control ratios, L/E (ratio of oligomycin-inhibited and non-coupled respiration) and ROUTINE flux control ratios, R/E (ratio of ROUTINE and non-coupled respiration) of the HepG2 cells treated with different PPAR ligands (**on the left**) and with increasing concentrations of ciglitizone (**on the right**) (**p < 0.01 and *** *p* < 0.001).

This complicated derangement typically alters some respiratory control ratios. Intriguingly, in intact cells, the L (leak)/E (ETS) ratio (leak control ratio), calculated according to both the Gnaiger [30] and Brand and Nicholls [31] methods, was significantly increased by the drug treatments, specifically: clofibrate +50% with respect to the control, *p* < 0.001, gemfibrozil: +33%, *p* < 0.01; bezafibrate: +25%, *p* = 0.01; and ciglitizone 1, 10, 20, 50 µmol: +50%, +67%, +116%, and +167%, respectively (Figure 7). Most importantly, in the intact cells, the inverse L/E (state 3u/state 4o) ratio, defined as cell RCR, analogous to the RCR of isolated mitochondria, confirmed a serious drug dyscoupling mitochondrial derangement. Moreover, the increase in R/E (routine control ratio, according to Gnaiger) depended on both a limited maximal oxidative capacity and an apparent concomitant increase in routine respiration.

**Figure 8:**
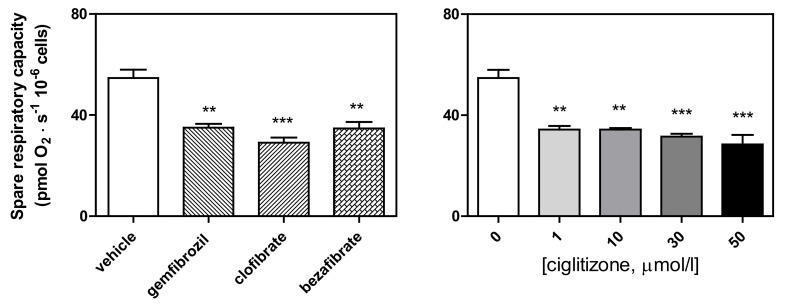
Spare respiratory capacity (calculated by subtracting the basal respiration from the maximal respiration in the presence of ATP) of the HepG2 cells treated with different PPAR ligands (** *p* < 0.01, and *** *p* < 0.001).

Finally, SRC, calculated according to the Brand and Nicholls method [31] as ETS respiration minus routine respiration, or the E–R capacity factor according to Gnaiger [35], was significantly reduced by all the drug treatments (ciglitizone: up to −47%, *p* < 0.001; gemfibrozil: −36%, *p* < 0.01; clofibric acid: −46%, *p* < 0.001; and bezafibrate: −35%, *p* < 0.01, respectively) (Figure 8).

### 3.3. Shotgun Proteomic Profiling of Differentiated HepG2 Cells by Label-Free nUPLC-MS^E^-Bioinformatics Analyses of Differentially Expressed Proteins

To better characterize the bioenergetic derangement induced by PPAR ligands, we performed a proteomic investigation by nano-ultra-performance liquid chromatography (nUPLC) coupled to the MS^E^ isotope-free shotgun profiling associated with bioinformatics analyses. For this experimental approach, we used the most potent energy-disrupting drug, ciglitizone, at 50 µm/L.

Approximately 300 proteins were identified in both CGZ-treated and untreated HepG2 cells. We quantified the proteome profile data for the differential expression analysis between two data sets. The label-free shotgun analysis in the samples enabled us to identify 67 differentially expressed proteins. To analyze the Gene Ontology (GO) classes and biologically relevant molecular pathways from our large-scale data, the list of differential proteins was subjected to further analysis by using two bioinformatics analysis tools, the Protein ANalysis THrough Evolutionary Relationships (PANTHER) classification system (http://www.pantherdb.org (accessed on 5 February 2020)) and Ingenuity Pathways Analysis (IPA; version 9.0, Ingenuity Systems, http://www.ingenuity.com). Using the PANTHER tool, modulated proteins are classified according to the family and subfamily to which they belong, as well as their respective molecular functions, biological processes and pathways.

In general terms, the most represented biological process modified by the drug treatment (35% of the total protein content) was related to cellular metabolism, in particular to primary metabolic processes, including nucleobase-containing compound metabolic processes, as well as protein, lipid and carbohydrate metabolic processes. Other drug-induced protein modifications were linked to various cellular processes important for cell communication and cell cycle regulation (24%), organization/biogenesis of cellular components (17%), specific cellular localization (8%) and developmental process (7%).

**Figure 9:**
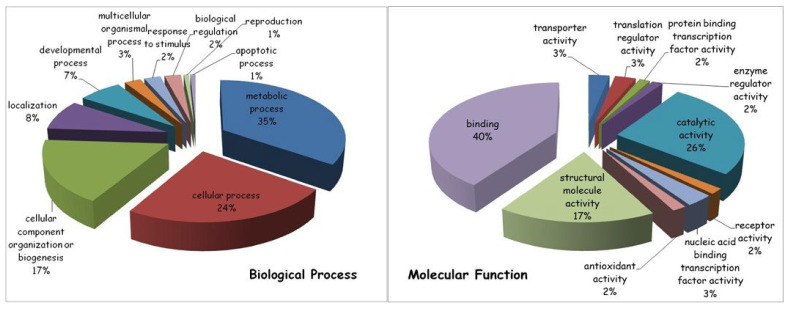
Gene ontology analysis of differentially expressed proteins (ciglitizone-treated cultures versus control cultures) using PANTHER online software. Proteins were classified according to their biological processes (**on the left**) and molecular functions (**on the right**).

Among the molecular functions, the majority of ciglitizone-modulated proteins (40%) were involved in binding activities, both of nucleic acids and of proteins, followed by catalytic (26%) and structural molecular (17%) activities (Figure 9).

Importantly, to study the possible physical and functional interactions among identified proteins, to understand mechanisms of drug-induced toxicity and to build a significant regulatory network, the list of differentially expressed proteins was subjected to pathway analysis using Ingenuity Pathway Analysis (IPA). By integrating the data set of ciglitizone-modulated proteins, the IPA software generated a tox list, which is a list of molecules involved in a particular type of toxicity, by correlating the entered data with a software database containing findings reported in the literature. Intriguingly, at the top of the tox list, IPA placed mitochondrial dysfunction by significantly associating several differentially expressed molecules, including two subunits of ATP synthase (ATP5A1, ATP5O), SOD2, PRDX3 and VDAC2, with mitochondrial derangement (*p* < 0.0003). The molecules ANXA5, HYOU1, LDHA, PPIA, PRDX3, SOD2 and VCP were connected to cell death and/or necrosis (*p* < 0.002), while other modulated proteins were linked to fatty acid metabolism (ACAT2, DHRS2, and ECHS1) (*p* < 0.005) and the alteration of the transmembrane potential of mitochondria and/or oxidative stress (PRDX3 and SOD2) (*p* < 0.01) (Table 1).

Other networks suggested by IPA (1–4 in Table 2) appear to be associated with important processes, ranging from infectious, developmental and neurological disorders to cell morphology, reproductive system development/function and lipid metabolism, all of which are related to mitochondria pathogenically.

Finally, yet importantly, some identified proteins analyzed by IPA generated a network that also appears to be involved in cancer pathophysiology.

**Figure 10:**
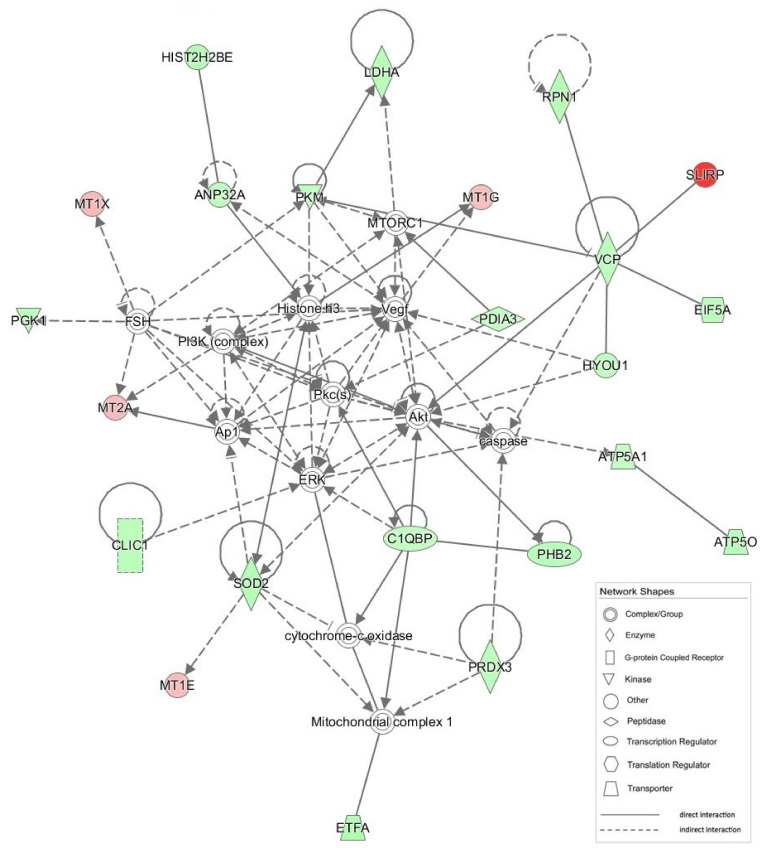
Graphical representation of the most significant IPA network (network # 1, score 51). Mitochondrial CI is in a pivotal position, for which a number of direct and indirect interactions are shown with different proteins modulated by CGZ.

The most significant IPA network (network #1; score = 51) is presented in Figure 10.

## 4. Discussion

It is well known that PPAR ligands (fibrates and thiazolidinediones), in addition to binding and activating Peroxisome Proliferator Activated Receptors, show many other interesting extra-peroxisomal biological activities [19,20]. In particular, they have been shown to inhibit NADH coenzyme Q reductase, thus influencing cellular respiration of numerous cell types to varying degrees [26]. The alteration of mitochondrial respiration caused by these drugs causes metabolic- and ROS-mediated stress that induces an arrest of the proliferation in human tumor cell lines and promotes a differentiation process which seems to be tightly correlated with the level of mitochondrial inhibition [24,25,26,27]. Interestingly, inhibition of C1 leads to the metabolic consequences typical of PPAR ligands pharmacological activities (hypolipidemic and hypoglycemic effects) [19,20] and this could, in turn, explain some of their toxic effects (rabdomyolisis, acute liver failure) already widely reported [14,15,16,17,18,19,20,21,22,23]. 

However, the molecular picture of this intriguing bioenergetic derangement has not yet been well defined. 

In our opinion, conducting in-depth analysis of drug–mitochondria interactions could provide new information on some intriguing aspects related to the pathophysiology of both oxidative metabolism and, most importantly, cellular oxygen homeostasis.

To this end, the complex interaction of mitochondrial ERC with so-called PPAR ligands was re-evaluated in the present study.

The HRR data underlined that mitochondrial derangement induced by different PPAR ligands was more complex than is shown through a simple spectrophotometric approach [27]. In this setting, it was evident early on that the CI inhibition of ERC by different PPAR ligands with a concomitant impairment of NADH oxidation was an epiphenomenon of larger and more complex mitochondrial dysfunction with important implications for cellular metabolism and target organ function. Finally, the damage in permeabilized and intact cells exhibited some significant differences.

In fact, in the permeabilized cells, the tested drugs significantly inhibited CI, and this effect was also associated with significant dysfunction in respiratory ETS capacity. This last piece of experimental evidence highlighted the potential reduction in the reserve capacity for oxidative phosphorylation caused by different drugs. However, this drug-induced dysfunction also provoked a significant alteration in RCR, which is considered the best general measure of mitochondrial function in permeabilized cells [29,31,35].

Finally, this set of experiments confirmed that ciglitizone, a PPAR-γ ligand, was the most potent energy disruptor, considering its toxicity index calculated as the inhibition of O_2_ consumption/drug concentration.

Interestingly, HRR performed on intact HepG2 cells highlighted a partially different picture of drug-induced mitochondrial derangement than that of the permeabilized cells. Specifically, while the ETS capacity showed a similar iatrogenic reduction in intact cells, leak respiration induced by oligomycin exhibited a paradoxical increase in oxygen consumption in the drug-treated cell cultures with respect to the controls. These contrasting data seemed to eliminate the possibility of a drug-induced uncoupling/dyscoupling effect related to proton leak and/or slip and cation cycling by the PPAR ligands. These paradoxical data drove us to consider the increase in oxygen consumption recorded in intact cells following oligomycin treatment, as it related to changes in oxygen bioavailability/utilization due to both an increase in or induction of various cellular enzymatic activities (i.e., oxygenases and oxidases, such as the induction of the cytochrome P450 family, which has a role in the disposal of these molecules) and in ROS production by altered electron flux in the ERC. These mechanisms seem to be confirmed by the increase in ROX and, moreover, by the high levels of fluorescence in the drug-treated cells in culture stained with DCF-DA with respect to the controls. Hence, the modification of oxygen utilization, particularly in cell cultures treated with ciglitizone, jeopardized the inhibitory effect on CI in the intact cells during routine respiration.

Importantly, in the intact HepG2 cells, some fundamental indexes (RCR, L/E and R/E, coupled respiration, and SRC) related to the functioning of the ERC showed significant perturbations induced by the PPAR ligands. Most importantly, considering the peculiar ERC derangement induced by these drugs, the true meaning of some flux control ratio indexes, which are usually adopted, seems to be misleading. All these considerations suggest that a prudent approach be used in evaluating an acquired mitochondrial derangement by simply considering control flux ratio indexes.

All the data seemed to show a significant limitation of respiratory capacity by defects in substrate oxidation and/or complexes of the ETS. Interestingly, the SRC measured in the HepG2 cells incubated with these PPAR ligands confirmed a significant perturbation of oxidative phosphorylation. This perturbation also seems to be associated with a significant production of reactive oxygen species, as shown by the DCF-DA study.

Overall, these data not only confirm and augment previous studies but also stress the significance of designing functional studies on mitochondrial pharmacology by planning a dual approach, at least (isolated mitochondria/permeabilized cells and intact cells) to limit the disadvantages of the single method that too often shows results subject to ambiguous interpretation.

Importantly, shotgun proteomic analysis permitted us to substantiate and deepen our understanding of the mitochondrial derangement, as highlighted by the HRR data showing, in drug-treated cells, a proteome modification linked to mitochondrial dysfunction, metabolic alteration and oxidative stress. In particular, among the downregulated proteins identified in this study and associated with mitochondrial functions, we cited the voltage-dependent anion-selective channel protein (VDAC2), the electron transfer flavoprotein ETFA and two subunits of ATP synthase (ATP5A1 and ATP5O), as well as several proteins with antioxidant functions localized in the mitochondrial matrix (SOD2 and PRDX3) and, moreover, several enzymes, such as ECHS1, which regulates fatty acid metabolism and is an essential factor in tumor development [38]. Interestingly, our proteomic data also revealed the downregulation of p32, also known as C1QBP (complement 1q-binding protein), a protein that has been detected in many different subcellular compartments, such as on the cell surface [39] and mitochondria [40], in CGZ-treated cells, associated with a wide spectrum of mitochondrial and non-mitochondrial functions.

Specifically, p32 has a crucial role in modulating tumor metabolism, particularly with regard to the regulation of the balance between OXPHOS and glycolysis, as shown by Fogal et al. [41]. Interestingly, p32 is involved in the synthesis of the mitochondrial-DNA-encoded OXPHOS polypeptides, directly influencing the expression levels of the CI 20-kDa and 30-kDa subunits and subunits I (COX1) and II (COX2) of CIV, thus indirectly affecting the amounts of other subunits, including subunits encoded by the nuclear genome. This in turn conceivably alters ROS and ATP production, which can influence mitochondrial permeability transition pore opening [42] a critical event in mitochondria-mediated cell death.

Therefore, considering the important metabolic effects of p32 protein, its marked reduction observed in the CGZ-treated cells seems to be in agreement with all the reported mitochondrial and metabolic alterations, as well as the significant perturbation of oxidative phosphorylation and the shift from OXPHOS to glycolysis, accompanied by an increase in lactate production and elevated glucose consumption, described in our previous studies [27]. Furthermore, other metabolic alterations induced by PPAR ligands, including the marked increase in pyruvate and alanine levels, which indicate NADH-induced partial inhibition of pyruvate dehydrogenase, are correlated with the ability of p32 to directly or indirectly bind to the PDH complex and regulate its activity [43].

Altogether, these proteomic findings reveal a picture of mitochondrial perturbation substantially in accord with that emerging from the HRR data, displaying protein alterations at the level of all the mitochondrial compartments, several of which directly correlate to respiratory chain activity, with an evident strong effect on mitochondrial bioenergetics.

Finally, it should be stressed that the data seem to indicate that, at therapeutic concentrations, PPAR ligands induce a partial derangement of mitochondrial NADH oxidation, but this derangement is enhanced by pharmacokinetic and/or pharmacodynamic interactions, as already dramatically shown in various clinical settings [44,45,46,47,48]. Moreover, it should be noted that small changes in cellular respiration and/or small derangements in respiratory control reflect significant mitochondrial damage, such as important alterations to the mitochondrial proteome and/or mtDNA, often associated with remarkable impairment to the mitochondrial signaling cascade.

## 5. Conclusions

Discussing all aspects of the important relationship that exists between mitochondria and cancer is quite difficult. However, it is possible to stress some aspects that highlight the potential translational applications of these intriguing pathophysiological links.

In our study, the mitochondrial bioenergetic damage induced by various PPAR ligands was evaluated, above all in the light of the intriguing pharmacotoxicological profile of thiazolidinediones (PPAR gamma ligands), which showed a pleomorphism of biological activities throughout their clinical history, accurately re-stated by Edwin Gale in its exhaustive review [49]. The profile ranges from the well-known insulin sensitizer effect to the debated oncosuppressor/oncopromoter activities and passing from significant therapeutic/toxic effects at the level of the heart, muscles, liver and kidneys [22,23,25,50,51,52,53,54]. Similarly, from a molecular point of view, there is a tangled pathophysiological role for these molecules (ERC disruptors, cyt P450 inducers, PPAR ligands, auxin-like activities, acute inhibitors of mitochondrial pyruvate carrier, and allosteric effectors of human hemoglobin) [22,23,24,25,50,51,52]. Delineating the drug–mitochondria interaction could be useful not only to clarify PPAR ligand pharmacotoxicology but also, more generally, to define mitochondrial pharmacology and its interrelationship with cellular homeostasis.

The evidence of a significant reduction in the respiratory control ratio, spare respiratory capacity, coupling efficiency together with a serious oxidative stress and structural damage of mitochondria inducted by fibrates and thiazolidinediones may explain some complicated aspects of their debated therapeutic index [55,56] and justify a push to consider these organelles as sensible “detectors” of potential/possible side effects.

The pathogenetic role of drug-induced alteration of the bioavailability, release and use of oxygen is evident. However, studying this iatrogenic derangement may help illuminate some obscure molecular mechanisms potentially related to the pathophysiology of aging and cancer. Finally, iatrogenic imbalance could be a useful context in which to explain possible/potential mitochondrial therapeutic strategies.

## Figures and Tables

**Table 1:** Ingenuity Pathway Analysis of the top toxic pathways in the HepG2 cells treated with ciglitizone.

Tox List	Proteins	*p* Value
Mitochondrial dysfunction	ATP5A1, ATP50, PRDX3, SOD2, VDAC2	2.74 × 10^4^
Cell death	ANXA5, HYOU1, LDHA, PPIA, PRDX3, SOD2, VCP	1.51 × 10^3^
Fatty acid metabolism	ACAT2, DHRS2, ECHS1	4.62 × 10^3^
Alteration transmembrane potential of mitochondria and mitochondrial membrane	PRDX3, SOD2	1.27 × 10^2^
Oxidative stress	PRDX3, SOD2	1.58 × 10^2^

**Table 2:** The top five biofunctions with their respective IPA scores.

ID	Molecules in Network	Score	Focus Molecules	Top Diseases and Functions
1	Akt, **ANP32A**, Ap1, **ATP5A1**, **ATP5O**, **C1QBP**, caspase, **CLIC1**, cytocrome-c oxidase, **EIF5A**, ERK, **ETFA**, FSH, **HIST2H2BE**, Histone H3, **HYOU1**, **LDHA**, Mitochondrial complex 1, **MT1E**, **MT1G**, **MT1X**, **MT2A**, MTORC1, **PDIA3**, **PGK1**, **PHB2**, PI3K (complex), Pkc(s), **PKM**, **PRDX3**, **RPN1**, **SLIRP**, **SOD2**, **VCP**, Vegf	51	23	Neurological Disease, Skeletal and Muscular Disorders, Hereditary Disorder
2	**ACAT2**, Actin, ADRB, **ANXA5**, CD3, **CFL1**, **DHRS2**, **DHX9**, ERK1/2, F Actin, **HNRNPK**, Hsp90, **KHDRBS1**, **KRT8**, MAP2K1/2, MATR3, **NACA**, **NCL**, PDGF BB, **PFN1**, **PPIA**, Rnr, **RPS12**, **RPS3A**, **RPSA**, Rsk, TCR, **TIMM13**, **TUBB**, **TUBB6**, **TUBB2A**, TUBB2B, tubulin complex, tubulin (family), **VDAC2**	48	21	Infectious Diseases, Developmental Disorder, Neurological Disease
3	AKT1, **ALDOC**, APBB3, APP, ATPAF2, ECE1, **ECHS1**, EED, EMG1, **ERP29**, FERMT2, FN1, GNRH2, HIRIP3, **HIST1H2BB**, **HIST1H2BD**, **HIS1H2BK**, **HIST1H2BM**, **HIST2H2BN**, **HIST2H2BF**, IGF2BP2, ITGA4, MAPK1, MBNL1, OSTF1, Ptk, RPLP2, **RPS12**, RPS16, **RPS4Y1**, SHOC2, TOMM22, VARS, YWHAZ, ZMAT3	20	11	Cell Morphology, Reproductive System Development and Function, Lipid Metabolism
4	26s Proteasome, Alpha actin, APLF, ARRB2, ASF1B, CDC34, CDKN2A, CUL1, CYP2EI, **DHX9**, GTF2E2, HIST1H2AG, **HIST1H2BA**, **HIST1H2BH**, **HIST1H2BJ**, **HIST1H2BL**, **HIST1H2BO**, **HIST3H2BB**, Histone h4, JMJD6, Jnk, MAP3K13, NFkB (complex), P38 MAPK, PAG1, PARP10, PTGES, Ras, **RBMXL2**, RNA polymerase II, RPLP2, **RPS12**, SIGIRR, TBP, ZMAT3	15	9	Cancer, Organismal Injury and Abnormalities, Reproductive System Disease
5	H2BFS, **MT1M**, PAN2	5	2	Psychological Disorders, Antimicrobial Response, Inflammatory Response

## Data Availability

Not applicable.

## Abbreviations

CI—Complex I; CII—Complex II; CIII—Complex III; CIV—Complex IV; ETS—electron transfer system; ERC—electron respiratory chain; FCCP—carbonyl cyanide p-trifluoromethoxyphenylhydrazone; RCR—respiratory control ratio (state 3/state 4); ROS—Reactive Oxygen Species; ROX—Residual oxygen consumption; UCR—uncoupling control ratio; state 3u—uncoupled state; state 4o—state 4 oligomycin; SRC—spare respiratory capacity.
